# Exercise as a Therapeutic Intervention in Gestational Diabetes Mellitus

**DOI:** 10.3390/endocrines2020007

**Published:** 2021-03-26

**Authors:** Konstantina Dipla, Andreas Zafeiridis, Gesthimani Mintziori, Afroditi K. Boutou, Dimitrios G. Goulis, Anthony C. Hackney

**Affiliations:** 1 Laboratory of Exercise Physiology and Biochemistry, Department of Physical Education and Sports Science at Serres, Aristotle University of Thessaloniki, 621 22 Serres, Greece;; 2 Unit of Reproductive Endocrinology, First Department of Obstetrics and Gynecology, School of Medicine, Aristotle University of Thessaloniki, “Papageorgiou” General Hospital, 56429 Thessaloniki, Greece;; 3 Department of Respiratory Medicine, “G. Papanikolaou” General Hospital, 57010 Exohi, Thessaloniki, Greece;; 4 Department of Exercise & Sport Science, Department of Nutrition, University of North Carolina, Chapel Hill, North Carolina, NC 27599-8700, USA;

**Keywords:** exercise endocrinology, pregnancy, exercise physiology, exercise, pregnancy endocrinology, diabetes, hormones, women’s health

## Abstract

Gestational Diabetes Mellitus (GDM) is defined as any degree of glucose intolerance with onset or first recognition during pregnancy. Regular exercise is important for a healthy pregnancy and can lower the risk of developing GDM. For women with GDM, exercise is safe and can affect the pregnancy outcomes beneficially. A single exercise bout increases skeletal muscle glucose uptake, minimizing hyperglycemia. Regular exercise training promotes mitochondrial biogenesis, improves oxidative capacity, enhances insulin sensitivity and vascular function, and reduces systemic inflammation. Exercise may also aid in lowering the insulin dose in insulin-treated pregnant women. Despite these benefits, women with GDM are usually inactive or have poor participation in exercise training. Attractive individualized exercise programs that will increase adherence and result in optimal maternal and offspring benefits are needed. However, as women with GDM have a unique physiology, more attention is required during exercise prescription. This review (i) summarizes the cardiovascular and metabolic adaptations due to pregnancy and outlines the mechanisms through which exercise can improve glycemic control and overall health in insulin resistance states, (ii) presents the pathophysiological alterations induced by GDM that affect exercise responses, and (iii) highlights cardinal points of an exercise program for women with GDM.

## 1. Introduction

Gestational Diabetes Mellitus (GDM) is a distinct subcategory of Diabetes Mellitus, defined as any degree of glucose intolerance with onset or first recognition during pregnancy [1,2]. GDM may affect nearly 5–15% of pregnancies worldwide, though ethnicity seems to also play an important role in its prevalence [3–5]. Well recognized risk factors for developing GDM include being overweight or obese, excessive weight gain during pregnancy, family history of GDM or Diabetes Mellitus type 2 (T2DM), high parity and advanced maternal age. Other modifiable risk factors include increased psychological stress, use of antidepressant and psychotropic medications, smoking, and poor sleep habits [6–8].

During pregnancy, placental hormones, such as human placental lactogen and placental growth hormone, cause a progressive development of insulin resistance in order to provide the fetus with the adequate amount of glucose. As a response to this, the number of maternal β-cells increases, and insulin synthesis and secretion increase in parallel. In women with GDM, the increased insulin resistance, together with a relative inability of the pancreatic β-cells to adapt to the increased needs, are suggested as the main pathophysiological events leading to glucose intolerance and hyperglycemia [9].

Complications of GDM include preeclampsia, macrosomia, neonatal hypoglycemia, large for gestational age offspring and an increased risk for caesarean delivery [10]. Women with a personal history of GDM also have an increased risk for developing T2DM later in life [10]. Besides maternal complications, the offspring of mothers with GDM are at increased risk of developing various diseases in later life. Although fetal programming (i.e., how embryonic/fetal environment determines responses that carry into adulthood and predisposes to certain postnatal diseases) is still under investigation, GDM seems to be a disease that may directly (and indirectly through intermediate outcomes) lead to T2DM, hypertension, obesity, and dyslipidemia in late childhood and adulthood [9,10].

## 2. Exercise in Pregnancy and Gestational Diabetes

Regular exercise is important for a healthy pregnancy. The American College of Obstetricians and Gynecologists’ (ACOG) recommendations indicate that pregnant women should be encouraged to commence or to continue their exercise training during pregnancy [11]. The recent guidelines (ACOG 2020) for exercise in pregnancy provide specific recommendations for (a) aerobic exercise in women who were recreationally active before pregnancy and those who were highly trained, for women who were sedentary, and those with obesity, and (b) resistance exercise (weight limits) based on previous experience with an emphasis on individualized exercise prescription [12]. The role of physical activity and exercise in the prevention of pregnancy-induced complications is also highlighted. However, the guidelines are aimed at women with uncomplicated pregnancies. For women with GDM, the American Diabetes Association recommends that they should exercise for approximately 30 min on most days to improve their glycemic control [13]. However, specific exercise guidelines for GDM are currently not available.

Previous investigations have shown that women with GDM who systematically participated in exercise programs during their pregnancy (2–7 days per week, for 30–60 min per session) had favorable results in fasting blood glucose and post-prandial glycemia and in fetal outcomes (lower birth weight) [14–18]. In some cases, exercise even delayed treatment with insulin [19]. Exercise is safe, with no reports showing increased maternal or neonatal complications in GDM [14]. However, women with GDM, like patients with T2DM [20], are usually inactive and have poor participation in exercise training programs. Thus, appropriate individualized exercise programs that will benefit maternal and offspring metabolic adaptations, but will be attractive and increase adherence, are still needed. As women with GDM represent a population with a unique physiology, they require more attention and monitoring during exercise. The altered responses of insulin-antagonistic hormones and the glucose fluctuations that exist due to obesity and hyperglycemia may modify the acute responses to exercise and exercise tolerance. In insulin treated GDM patients, the increasing insulin resistance during pregnancy causes specific challenges for exercise prescription, as adjustments on insulin doses and carbohydrate intake are required to prevent hypoglycemia during and after training.

In light of the prior findings, this review aims to summarize the physiological adaptations due to pregnancy and to update the mechanisms through which exercise can improve glycemic control and overall health in insulin resistance states. In addition, the pathophysiological alterations induced by GDM that can affect the exercise responses will be presented. Finally, the key points in the design of an exercise program for women with GDM will be highlighted.

## 3. The Role of Exercise in Improving Glycemic Control and Overall Health in Insulin Resistance States

Several studies highlight the beneficial effect of exercise in the prevention and treatment of GDM; however, the exact mechanisms underlying the exercise-induced benefits in pregnancy complicated by diabetes have been extrapolated mostly from human studies in prediabetes or T2DM and experimental data in animal models. This section summarizes how acute and chronic exercise can improve glycemic control and enhance overall health in normoglycemic and in insulin resistance states.

### 3.1. The Acute Effects of Exercise in Facilitating Greater Glucose Uptake by the Skeletal Muscles

Exercise significantly increases muscle glucose uptake (approximately a 100 fold increase from resting levels has been reported) [21]. This augmented glucose uptake by the skeletal muscle cells during contraction is the result of the greater glucose delivery, greater membrane permeability to glucose, and enhanced intracellular metabolism of glucose [22–25]. During exercise, hepatic glucose production increases to ensure its sufficient plasma concentrations. Although during exercise pancreatic insulin output decreases, there is enough insulin to stimulate a greater microvascular perfusion at the skeletal muscle level [26,27]. Thus, the increase in blood flow and capillary recruitment during exercise facilitate a greater glucose delivery to the contracting skeletal muscles [27]. The rapid translocation of glucose transporters type 4 (GLUT4) from intracellular storage sites to the sarcolemma and t-tubules is considered the fundamental event for promoting glucose diffusion from the interstitial space to the cytoplasm of the skeletal myocyte. During the initial exercise stages, glucose phosphorylation to glucose 6-phosphate by hexokinase is an important step initiating muscle glucose metabolism [27,28].

**Figure 1.**
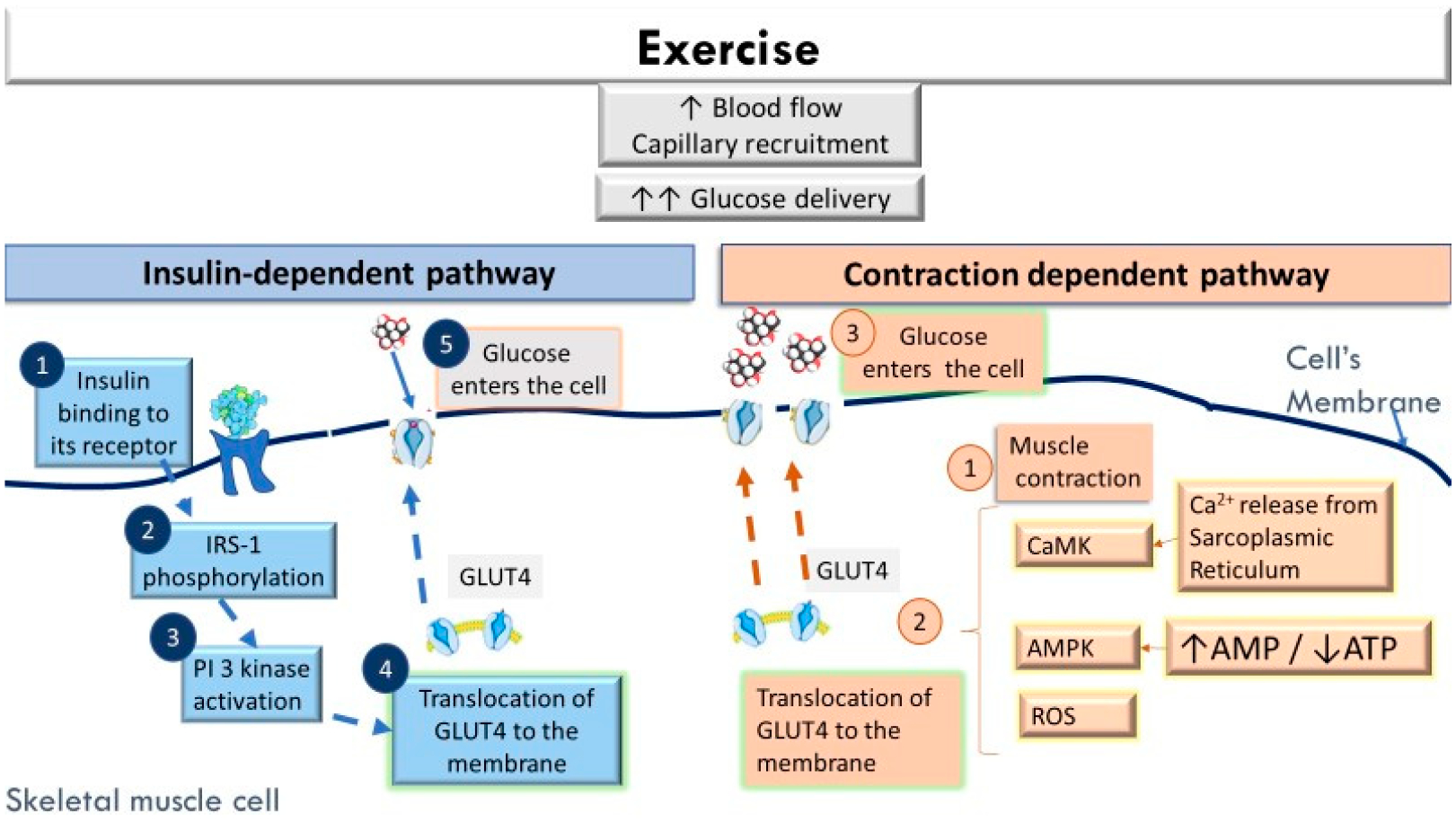
During exercise, the increase in skeletal muscle blood flow and capillary recruitment increase glucose delivery. Glucose uptake by the skeletal muscle cell is facilitated by two separate pathways: (i) an insulin-dependent pathway (which begins with insulin binding to its receptor (IRS), followed by its phosphorylation (1–2). In turn, the phosphoinositide 3-kinase (PI3K) pathway (3) is activated, and through a series of reactions, the translocation of GLUT4 to the cell membrane is stimulated (4)) and (ii) a contraction dependent pathway (which is mediated by several cellular events related to muscle contraction per se (1), such as the release of Ca^2+^ by the sarcoplasmic reticulum to be used for muscle contraction and the activation the Ca^2+^/calmodulin-dependent protein kinase (CaMK), the reduction in the ATP/ADP ratio and the activation of AMP-protein kinase (AMPK), the transient increase in oxidative stress), which triggers the translocation of GLUT4 to the cell membrane. Although in insulin resistance states the insulin dependent pathway is dysfunctional, the contraction dependent pathway seems to remain intact. Therefore, exercise can promote greater glucose uptake and reduce hyperglycemia. A greater glucose uptake (vs. pre-exercise) remains in the post-exercise recovery period (depending on the characteristics of the exercise session). ROS: Reactive oxygen species; GLUT4: Glucose transporters 4.

Specifically, during exercise, the redistribution of GLUT4 glucose transporters from the cell interior to the cell surface facilitates glucose uptake by two separate, but additive pathways (Figure 1) [22,24–26,29–32]. The first pathway is the insulin-dependent pathway that begins with insulin binding to its receptor (IRS) and the phosphorylation of intracellular tyrosine residues in IRS. This phosphorylation activates the phosphoinositide 3-kinase (PI3K) pathway, which in turn catalyzes the formation of phosphatidylinositol (3,4,5)-trisphosphate (PI3P). PI3P activates the protein kinase B isoform (Akt) and the protein kinase C (aPKC) and triggers the translocation of GLUT4 to the cell membrane [33]. The second pathway that facilitates glucose uptake during exercise is a non-insulin dependent pathway which is mediated by several cellular events related to muscle contraction per se [22,25,28,29]. Potential signals mediating this exercise-induced GLUT4 translocation include: (a) the release of Ca^2+^ by the sarcoplasmic reticulum to be used for muscle contraction, which causes an increase in intracellular Ca^2+^ concentration and activates the Ca^2+^/calmodulin-dependent protein kinase (CaMK), (b) the reduction in the ATP/ADP ratio and the activation of AMP-protein kinase (AMPK), and (c) the transient increase in oxidative stress [28,34,35]. Additional exercise-induced mechanisms that have been described include increased activity of endothelial nitric oxide, the activation of mitogen-activated protein kinase (MAPK), the activation of protein kinase C (PKC), bradykinin, hypoxia, and the increase in muscle temperature along with the mechanical deformation during muscle contraction [21,36,37]. The synergistic action of the above mechanisms stimulates an effective translocation of GLUT4 to the cell’s membrane during muscle contraction and relaxation [28].

During the post-exercise period, there is a persistent increase in insulin-independent glucose uptake for approximately 2–3 h [37]. An enhanced muscle and whole-body insulin sensitivity, however, can persist for up to 24–48 h, depending on the type and duration of the exercise stimulus [27,33,38–40]. For this reason, the frequency of exercise is an important component to consider when designing an exercise program for insulin resistant individuals. Insulin resistance results in a dysfunctional insulin-stimulated GLUT4 pathway in the skeletal muscle of obese, T2DM, and GDM patients [41,42]; however, the non-insulin dependent pathway seems to remain intact in obesity and insulin resistant stages [22,43,44]. Therefore, exercise can provide an alternate pathway to increase glucose uptake and lessen blood hyperglycemia in insulin resistant individuals. In addition, the release of myokines during exercise, such as irisin, interleukin (IL)-15, IL-7, brain-derived neurotrophic factor (BDNF), and myonectin, also plays an anti-inflammatory role [32].

### 3.2. The Long-Term Effects of Regular Exercise on Glycemic Control and Overall Health in Insulin Resistance States

The repeated, transient increases in *GLUT* transcription during an acute exercise bout lead to a gradual increase in GLUT4 protein after short-term exercise training [23]. Increased expression and protein content of GLUT4 have been described in response to training in T2DM [28,45,46]. In addition to the increase in GLUT4 expression and protein levels, the increases in muscle glycogen synthase, glycogen content, and hexokinase II all contribute to increased insulin sensitivity in response to training [47]. Notably, regular exercise also promotes adaptations (in blood vessels and muscle cells) that facilitate greater delivery, uptake, and utilization of glucose and nutrients during a physical task, which are presented in more detail below.

Exercise training improves insulin-mediated increases in capillary recruitment in combination with augmented muscle glucose uptake [27,48]. Studies in diabetic humans showed that exercise training increased skeletal muscle vasculature content and limb blood flow and improved effective blood flow/oxygen delivery [49]. At the skeletal muscle level, an important chronic adaptation to exercise (mainly aerobic) is mitochondrial biogenesis [50–52]. The increased activity of CaMK and AMPK kinases during an acute exercise bout activates peroxisome proliferator-activated receptor gamma coactivator 1-alpha (PGC-1*α*) [53,54]. Through the processes of fusion (merging of the outer and the inner mitochondrial membranes of two originally distinct mitochondria) and fission (fragmentation of mitochondria), the mitochondria become more capable to overcome energetic challenges [55,56]. Furthermore, dysfunctional mitochondrial sites are removed through mitophagy [55,56], reducing the negative effects of oxidative stress on mitochondrial DNA. These exercise-induced changes in mitochondrial dynamics seem to be specific to muscle fiber type, as exercise induces mitochondrial elongation in oxidative fibers (Type I, IIA) and more fused mitochondria in glycolytic (Type IIX, IIB) fibers [50,51]. As obesity and diabetes reduce mitochondrial biogenesis in skeletal muscle and increase the accumulation of dysfunctional cellular organelles, exercise training can attenuate mitochondrial dysfunction, allowing the mitochondria to maintain the balance between mitochondrial dynamics and mitophagy [57–60]. These chronic training adaptations increase the ability for oxidative metabolism (providing energy for a longer exercise time) and result in less accumulation of fatty acids and lipid byproducts in blood caused by the diabetic phenotype [61,62].

A combination of caloric restriction and exercise can also reduce obesity-associated inflammation [63]. Animal data (in high-fat diet-fed mice) suggest that exercise alleviates inflammation in the adipose tissue by inducing a macrophage phenotype switch from M1- to M2-macrophages and by suppressing the infiltration of inflammatory macrophages in adipose tissue [64,65]. Data in diabetic mice also suggest that exercise alleviates the inflammation and oxidative stress of perivascular adipose tissue, contributing to improvements in endothelial vascular function [66]. Exercise training can also improve brain function in insulin resistance states. In diabetes, advanced glycation end-products (AGEs) are considered to contribute to the development of alterations in cerebral capillaries, leading to a disruption of the blood–brain barrier [67]. Briefly, AGEs (macromolecules formed by a process known as glycation that occurs when glucose reacts with proteins, lipids, and nucleic acids after chronic exposure to hyperglycemia) are widely distributed in the body and their accumulation can affect the vascular basement membrane and extracellular matrices [68]. The accumulation of AGEs in the brain upregulates inflammatory cytokines and adhesion molecules, leading to chronic inflammation, amplified oxidative stress, and cerebral microvascular endothelial cell damage [67,69]. While AGEs increase during normal aging [70], their formation accelerates in diabetes, and this has been considered one of the possible links of diabetes and the development of cognitive impairments [69]. Regular exercise promotes neuroplasticity, improves metabolic efficiency, and reduces oxidative stress; thus, exercise training can beneficially affect brain function [71–73]. Although the exact mechanisms of these adaptations and the role of exercise in reducing AGEs are still under investigation, the release of neurotrophic factors during repeated exercise stimuli promotes neurogenesis, increases capillarization, decreases oxidative damage, and increases proteolytic degradation by proteasome and neprilysin [73]. These changes lead to a decreased accumulation of carbonyls and amyloid beta-proteins and can possibly improve memory. Collectively, it appears that exercise-induced alterations of the redox state are important means by which exercise benefits brain function, increases oxidative stress tolerance, and promotes faster recovery from oxidative stress [72,73]. Exercise training can also modify hepatic gene expression and hepatic pathways related to metabolic disease [74]. Regular exercise has been shown to reduce liver fat content, independently of weight loss (for a review, see [75]). This effect of exercise on the liver may be related to the improvement in insulin sensitivity and lipid metabolism; however, the exact impact of exercise on insulin-stimulated hepatic insulin sensitivity remains unclear. In recent years, the influence of exercise training on the dysbiotic gut microbiota associated with obesity and insulin resistance has also been investigated. Exercise training improved gut microbiota profiles and reduced endotoxemia in individuals with insulin resistance or diabetes [76]. However, the exact mechanisms underlying the potential cross link between exercise and the diabetic gut microbiota require further studies.

Importantly, the role of exercise training in preventing/minimizing the negative effects of maternal obesity and diabetic exposure in the offspring of GDM (i.e., reduce obesity, insulin resistance, and hepatic steatosis in the offspring) has been highlighted by recent studies in animals. Specifically, gestational exercise in GDM animals reduced offspring hepatic triglycerides accumulation and improved liver mitochondrial respiratory capacity [77]. These beneficial effects were preserved even after the cessation of the exercise training program. In rats, maternal exercise attenuated the lower skeletal muscle glucose uptake and insulin secretion caused by paternal obesity in female adult offspring [78]. These studies show the importance of gestational exercise as a preventive tool against maternal diet-induced metabolic alterations.

In summary, exercise can play an important role in improving metabolic disturbances and mitochondrial quality, reducing inflammation and chronic oxidative stress induced by obesity and hyperglycemia, and enhancing overall health in insulin resistance states.

## 4. Exercise Prescription in Gestational Diabetes Mellitus

Describing the physiological changes in pregnancy is important for understanding the acute and chronic adaptations to exercise in pregnancy. Below, we present physiological/metabolic alterations of GDM that should be considered when designing the exercise program.

### 4.1. Acute Cardiovascular and Metabolic Adaptations to Exercise in Pregnant Women with Uncomplicated Pregnancies

#### 4.1.1. Physiological Alterations in Pregnancy

During pregnancy, several physiological, but also anatomical, changes occur to support the greater metabolic demands of the developing fetus. The uterus enlarges, there is an increase in vascularization, and blood flow to the placenta increases to meet the needs of the fetus. To accommodate these greater demands and altered metabolic status, the cardiovascular system of the woman must also undergo appropriate structural and hemodynamic adaptations [79]. Due to the upward displacement of the diaphragm (due to the growing fetus), the heart is pushed upward and rotated forward [80]. As early as at 4-weeks of gestation, the maternal heart rate increases beyond pre-pregnancy levels (via an increased sympathetic tone and/or a decreased parasympathetic) and progressively increases up to the third trimester when a plateau in heart rate is observed. Overall, a 20–25% increase in heart rate compared to pre-pregnancy levels occurs and contractility and stroke volume increase due to the greater myocardial preload [79]. As a result of the higher cardiac output, the heart undergoes remodeling. Left ventricular wall thickness and mass increase, and a reversible, physiological hypertrophy develops (non-pathological).

In the early gestational weeks, hormonal changes (i.e., increased estradiol, activation of the renin-angiotensin-aldosterone system, increased erythropoietin) result in water retention and hematological adaptations, such as increases in plasma and red blood cell volume. In addition, the increased progesterone, nitric oxide, relaxin, and prostaglandins lead to a systemic vasorelaxation (decreased systemic vascular resistance), and thus, a slight reduction in systemic blood pressure. Mean arterial blood pressure decreases by approximately 5 to 10 mmHg during the second trimester; however, as gestation progresses, blood pressure gradually increases to pre-pregnancy levels [79]. Water retention and gestation-dependent edema is a common finding in a large percentage of pregnant women. The hormonal changes and placenta ensure the delivery of glucose to the fetus during pregnancy. Briefly, the maternal production of glucose increases, while insulin resistance at the skeletal muscle level increases (especially during the second and third trimester) to facilitate greater glucose delivery to the developing fetus [14,81]. Even a normal pregnancy has been considered as a “diabetogenic state” because of the progressive rise in postprandial glucose and insulin levels in late gestation, a decrease in insulin-mediated glucose disposal (by approximately 50%), and a significant increase in insulin secretion (by 200–250%) to maintain euglycemia in the mother [82,83]. However, the early gestational period can also be considered as an anabolic condition because of the increase in maternal fat stores and the decrease in free fatty acid levels [82,83]. During late pregnancy, insulin lessens its ability to suppress whole-body lipolysis; thus, in the postprandial period, greater increases in fatty acids and greater hepatic glucose production are observed, and this effect is more pronounced in women with insulin resistance/GDM [82,84].

#### 4.1.2. Physiology of Exercise in Uncomplicated Pregnancies

The physiological changes during pregnancy affect some acute adaptations to exercise [79]. For example, even healthy women experience faster fatigue during pregnancy compared to their pre-pregnancy levels and a slight shortness of breath on exertion. Despite the greater cardiac output at rest, the maximal cardiac output during exercise is attained at lower work levels [85]. Oxygen uptake (VO_2_) and minute ventilation at resting conditions and during submaximal weight-supported exercise (walking, treadmill) are higher compared to the pre-pregnancy values. In fact, for weight bearing activities, VO_2_ increases in proportion to the maternal weight gain. For this reason, non-weight bearing activities (bicycle or water-based activities) might be more pleasant and tolerable in unfit pregnant women. Although resting heart rate is higher during pregnancy, maximal heart rate is lower at late pregnancy [79,85]. This translates to a lower heart rate reserve, which should be considered when designing the exercise program.

### 4.2. Metabolic, Neural, and Vascular Alterations that Can Affect the Responses to Exercise in Gestational Diabetes

The ability to perform exercise depends on the optimal oxygen delivery and utilization, which is a result of an interplay of multiple physiological functions and reflexes, such as pulmonary ventilation and gas exchange, cardiac output, blood flow and diffusion, skeletal muscle oxidative and force-generating capacity, as well as fatigue perception. In obesity and insulin resistance states, alterations have been described in the majority of these functions (for a review, see Nesti et al. [86]).

Accumulating evidence in non-pregnant individuals suggests that the blood pressure response to exercise is often exaggerated in obesity or insulin resistance (T2DM), in part, due to an exaggerated exercise pressor reflex [87–91]. An increased sympathetic stimulation can increase peripheral resistance and attenuate blood flow to the active muscles during exercise. Furthermore, the impaired cardiac autonomic function in insulin resistance/obesity [92,93] can result in unfavorable alterations in the heart rate at rest and during exercise.

Endothelial dysfunction is another well described phenomenon in insulin resistance/diabetes that can limit exercise performance. Although in women with GDM the metabolic insult is relatively short term (as diabetes develops during pregnancy), research findings suggest that even this short exposure to the hyperglycemic environment is sufficient to induce vascular dysfunction [94–96]. Macro- and microvascular dysfunction can induce changes in blood flow and impede exercise performance. A sluggish hyperemic response in the microvessels of the skeletal muscles in GDM has been reported, suggesting an inability of small vessels (i.e., terminal arterioles) to dilate in response to stimuli and/or a capillary rarefaction [96]. These microvascular alterations in GDM can hinder the fundamental pregnancy adaptations for vasodilatation and induce premature fatigue during exercise.

At the skeletal muscle level, obesity and diabetes can alter mitochondrial dynamics [53,59,97,98]. In women with GDM, a reduced capacity for muscle oxygen extraction during an arterial occlusion maneuver was reported (vs. women with uncomplicated pregnancies), implying a reduced muscle oxidative capacity [96]. Even when pregnant women with and without diabetes were matched for body mass index, the rate of oxygen uptake by the skeletal muscles (as assessed by near-infrared spectroscopy (NIRS) during an arterial occlusion) was still lower in women with GDM [96]. In accordance with that study, reduced skeletal muscle oxidative phosphorylation and disordered calcium homeostasis (as suggested by a 75% reduction in AMPK phosphorylation) in obese women with GDM were also shown [59]. The alterations in the balance between oxygen delivery and utilization as well as in the muscles’ metabolism may partially contribute to the blunted muscle oxygenation and exercise intolerance in women with GDM [96]. Besides the metabolic disturbances, blood flow dysregulation can also have profound effects on brain structure and vasculature. In fact, lower cerebral oxygenation (as assessed by NIRS) and increased fatigability during exercise have been described in non-pregnant adults with T2DM [99]. In GDM, blunted cortical oxygenation during exercise and a delayed recovery compared with women with uncomplicated pregnancies were reported [100]. Of note, women with GDM were able to maintain a lower average force output despite reaching similar fatigue levels as their control counterparts. These findings suggest a possible link of exercise intolerance with the reduced cerebral oxygenation/cortical activation during physiological stress in women with GDM that should be taken into consideration when designing the exercise program. Interestingly, the alterations in vascular parameters and muscle/cerebral oxygenation in women with GDM were not entirely reversed post-partum (6–9 months following delivery) [101], suggesting that special consideration in exercise prescription should also be given in the post-partum period.

### 4.3. Exercise Prescription Characteristics in Gestational Diabetes Mellitus

#### 4.3.1. Important Characteristics of the Exercise Program

As noted above, exercise enhances glucose uptake; however, this effect is short-lived (up to 24 h, depending on the exercise characteristics). Therefore, exercise specialists should aim to prescribe daily exercise sessions in GDM. For boosting insulin sensitivity, women should not stay for more than 2 days without exercise. Monitoring the exercise intensity is another important point to consider. For identifying the appropriate intensity, heart rate reserve is the preferred method over the percentage of maximal heart rate. In general, consideration should be given when choosing the appropriate training “zone” based on heart rate. As presented above, both obesity and insulin resistance can result in alterations in autonomic nervous system function. Thus, the heart rate responses to exercise might be altered. In some women, heart rate might be higher during light intensity exercise, while at high/maximal exercise, heart rate might be blunted [102]. For this reason, monitoring the rate of perceived exertion (RPE) is important. The perceived exertion should be at 12–14 (20-point scale; somewhat hard) on the Borg scale. Thus, both heart rate and RPE scale should be considered when monitoring the exercise intensity. The “talk test” is an alternative easy and practical tool for monitoring exercise intensity (the intensity is considered below the ventilatory threshold when the individual is able to freely converse without “pausing” her breath) [103].

Current ACOG guidelines suggest that women with uncomplicated pregnancies who were regularly exercising before pregnancy could engage in moderate to high intensity exercise [12]. In general, higher intensity and volume of exercise provide better benefits in glycemic control. However, for women with GDM, especially those that were sedentary before pregnancy and are overweight or obese, the first stages of exercise training should involve low intensity tasks. For the initial exercise sessions (during the first couple of weeks, depending on the participant), a short duration aerobic exercise session (12–15 min) is preferred in overweight/obese and unfit women without training experience, so that they become accustomed to exercising. As the training progresses, sessions >30 min of aerobic (fast walking, bicycling) or circuit training should be an ideal target [104]. In the first trimester of pregnancy, a duration of ≥38 min per day of moderate-intensity exercise or ≥264 min per week of moderate exercise has been recently suggested for preventing GDM [105]. To summarize, the first goal in the training program should be to increase the frequency, up to at least five times per week, and then increase the exercise session duration to at least 30 min. Finally, the intensity should gradually increase, as tolerated.

#### 4.3.2. Avoiding Exercise-Induced Hypoglycemia in Insulin Treated Women

The exogenous administration of insulin results in a lack of a decline in blood insulin during exercise and an inappropriate magnification of the effects of insulin on the liver and peripheral tissues [106]. It must be considered that during exercise the increase in blood flow can accelerate the absorption of the subcutaneous injected insulin, especially if insulin is injected at a site near the active muscles [106]. Thus, prolonged (>45 min) or high-volume exercise can result in hypoglycemia in insulin treated women with GDM if adjustments in insulin dose are not made. If the woman plans to exercise, appropriate reductions in insulin levels based on the characteristics of exercise (duration and intensity) should be made. Glucose should be monitored before the start of the exercise session; adequate caloric supplementation is important to minimize the hypoglycemia risk before, during and after exercise [107] depending on the insulin analogs utilized [108]. Additionally, postprandial walking (up to 2 h after the meal) can be beneficial in reducing post-prandial glucose levels [108]. Exercising late in the evening should be avoided in order to minimize the risk of nocturnal hypoglycemia.

#### 4.3.3. Which Type of Exercise Can Result in the Most Favorable Glycemic and Health Benefits?

Several studies in animals and humans have shown that aerobic exercise improves mitochondrial function, reduces oxidative stress and inflammation, improves vascular function, and stimulates angiogenesis. Thus, large muscle mass aerobic type activities (fast walking, stationary cycling, water aerobics) can improve aerobic fitness in pregnant women [12,109,110]. Furthermore, resistance exercise (light weights and elastic bands) can result in beneficial effects in muscle mass and stimulate greater glucose uptake. Muscle strengthening can minimize the risk for low back pain and falls [111].

**Figure 2.**
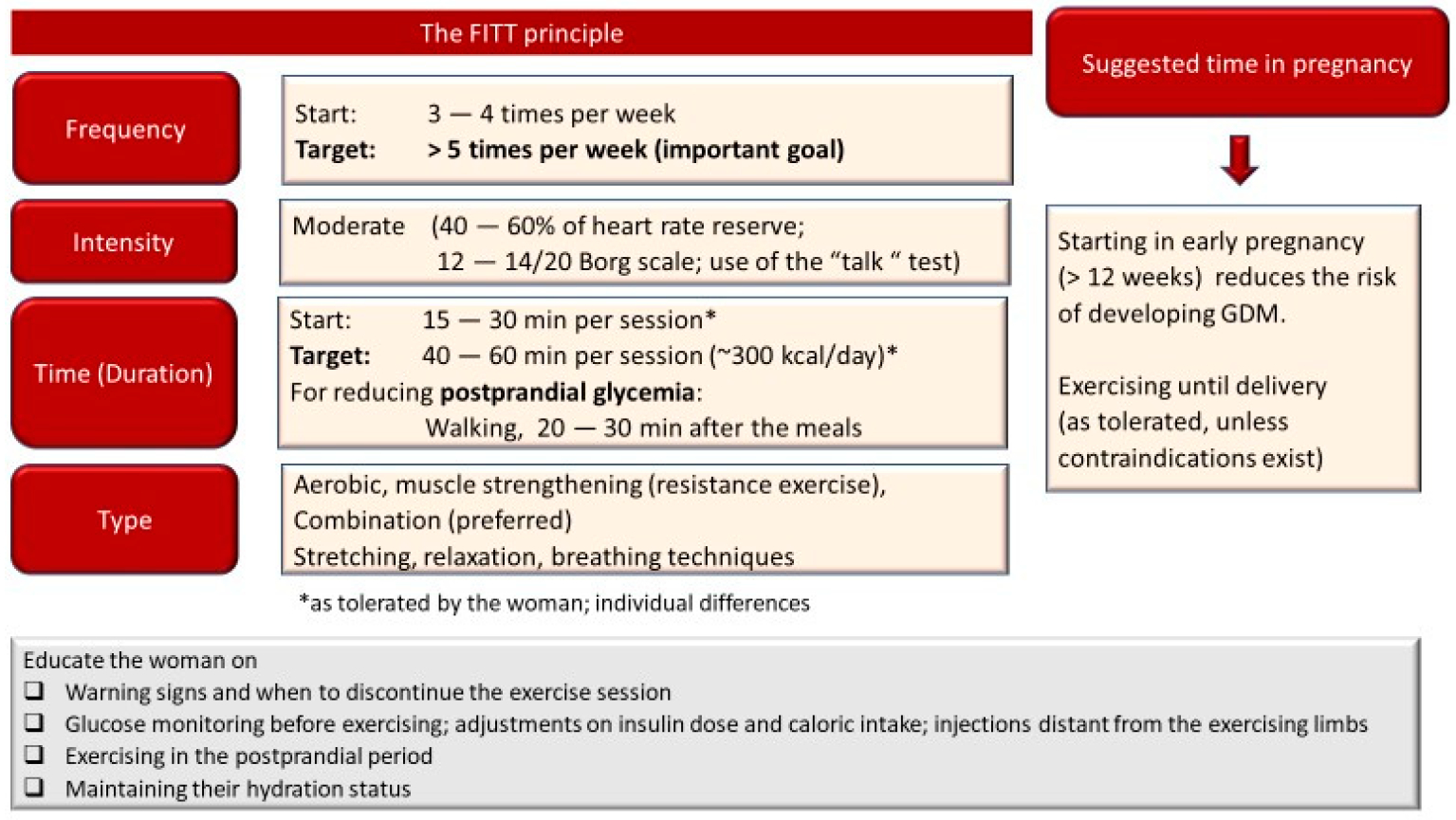
An example of an exercise program based on recommendations for pregnant women, with adjustments for women with gestational diabetes mellitus (GDM). FITT: frequency, intensity, time, and type of the exercise program.

Meta-analytic data by Huang et al. suggested that aerobic exercise reduced fasting and postprandial blood glucose and glycosylated hemoglobin levels in women with GDM [112]. Resistance exercise or combined aerobic and resistance exercise resulted in significant reductions in the required dosage of insulin in insulin-treated women with GDM. Combined training also reduced postprandial blood glucose [112]. Results from another meta-analysis also showed that exercising three times per week for 40–60 min at 65–75% of age-predicted maximum heart rate using cycling, walking or combined (circuit) training improved glycemic control in GDM patients and reduced the incidence of GDM in pregnant women with obesity [104]. In Figure 2, an example of an exercise program considering the recommendations for pregnant women and adjustments for women with GDM, and recommendations for improving glycemic control in patients with T2DM, is presented. Further studies that will specifically investigate the exercise characteristics and the effects of different exercise modalities to maximize benefits for GDM pregnancies are needed.

## 5. Conclusions

In conclusion, regular exercise during pregnancy results in beneficial effects in GDM. A single acute exercise bout can induce short-term glycemic control improvement. Regular exercise training during pregnancy promotes adaptations in the skeletal muscles, resulting in improved oxidative capacity, increased expression of proteins involved in mitochondrial biogenesis, enhanced lipid oxidation, and improved insulin sensitivity and glucose uptake. These adaptations, in turn, result in less inflammation and better vascular function in women with GDM. Thus, exercise has a positive impact on the metabolic profile and overall health of women with GDM. The participation of insulin-treated women with GDM in exercise training sessions may lower insulin doses. The exercise induced maternal benefits during pregnancy also induce positive effects in the later life of the mother and of the developing fetus. The efficacy of the exercise interventions depends on the characteristics of the exercise program. Exercise specialists should take into consideration the metabolic and vascular alterations induced by GDM, along with the considerations in pregnancy when designing the exercise program. Individualized exercise programs can markedly increase the adherence and the effectiveness of the exercise program for pregnant woman.
